# Early Effects of Sacubitril/Valsartan on Exercise Tolerance in Patients with Heart Failure with Reduced Ejection Fraction

**DOI:** 10.3390/jcm8020262

**Published:** 2019-02-20

**Authors:** Giuseppe Vitale, Giuseppe Romano, Antonino Di Franco, Giuseppa Caccamo, Cinzia Nugara, Laura Ajello, Salvo Storniolo, Silvia Sarullo, Valentina Agnese, Francesco Giallauria, Giuseppina Novo, Francesco Clemenza, Filippo M. Sarullo

**Affiliations:** 1 Cardiovascular Rehabilitation Unit, Buccheri La Ferla Fatebenefratelli Hospital, 90123 Palermo, Italy; giuseppevit@hotmail.com (G.V.); caccamo.giuseppa@libero.it (G.C.); cinzianugara@gmail.com (C.N.); 2 Department for the Treatment and Study of Cardiothoracic Diseases and Cardiothoracic Transplantation IRCCS – ISMETT (Istituto Mediterraneo per i Trapianti e Terapie ad Alta Specializzazione), 90127 Palermo, Italy; gromano@ismett.edu (G.R.); lajello1305@libero.it (L.A.); vagnese@ismett.edu (V.A.); fclemenza@ismett.edu (F.C.); 3 Department of Cardiothoracic Surgery, Weill Cornell Medicine, New York, NY 10065, USA; and2052@med.cornell.edu; 4 Biomedical Department of Internal Medicine and Specialities (DIBIMIS), University of Palermo – IRCSS Bonino Pulejo, 98124 Messina, Italy; 5 Cardiology Unit, University Hospital, Policlinico Paolo Giaccone, 90127 Palermo, Italy; salvo.storniolo@gmail.com (S.S.); silvia.sarullo@libero.it (S.S.); giuseppina.novo@unipa.it (G.N.); 6 Department of Translational Medical Sciences, Division of Internal Medicine, Metabolic and Cardiac Rehabilitation Unit, Federico II University, 80138 Naples, Italy; francesco.giallauria@unina.it

**Keywords:** heart failure, sacubitril/valsartan, cardiopulmonary test, exercise tolerance

## Abstract

Background. Sacubitril/valsartan in heart failure (HF) with reduced ejection fraction (HFrEF) was shown to be superior to enalapril in reducing the risk of death and hospitalization for HF. Our aim was to evaluate the cardiopulmonary effects of sacubitril/valsartan in patients with HFrEF. Methods. We conducted an observational study. Ninety-nine ambulatory patients with HFrEF underwent serial cardiopulmonary exercise tests (CPET) after initiation of sacubitril/valsartan in addition to recommended therapy. Results. At baseline, 37% of patients had New York Heart Association (NYHA) class III. After a median follow-up of 6.2 months (range 3–14.9 months) systolic blood pressure decreased from 117 ± 14 to 101 ± 12 mmHg (*p* < 0.0001), left ventricular ejection fraction (LVEF) increased from 27 ± 6 to 29.7 ± 7% (*p* < 0.0001), peak oxygen consumption (VO_2_) improved from 14.6 ± 3.3 (% of predicted = 53.8 ± 14.1) to 17.2 ± 4.7 mL/kg/min (% of predicted = 64.7 ± 17.8) (*p* < 0.0001), minute ventilation/carbon dioxide production relationship (VE/VCO_2_ Slope) decreased from 34.1 ± 6.3 to 31.7 ± 6.1 (*p* = 0.006), VO_2_ at anaerobic threshold increased from 11.3 ± 2.6 to 12.6 ± 3.5 mL/kg/min (*p* = 0.007), oxygen pulse increased from 11.5 ± 3.0 to 13.4 ± 4.3 mL/kg/min (*p* < 0.0001), and ∆VO_2_/∆Work increased from 9.2 ± 1.5 to 10.1 ± 1.8 mL/min/watt (*p* = 0.0002). Conclusion. Sacubitril/valsartan improved exercise tolerance, LVEF, peak VO_2_, and ventilatory efficiency at 6.2 months follow-up. Further studies are necessary to better clarify underlying mechanisms of this functional improvement.

## 1. Introduction

Combining renin-angiotensin-aldosterone system blockade with natriuretic peptide system enhancement may deliver functional benefits to patients with heart failure (HF) with reduced ejection fraction (HFrEF). In the PARADIGM-HF study, angiotensin receptor/neprilysin inhibitor sacubitril/valsartan was shown to be superior to enalapril in reducing the risk of death and hospitalization for HF [1]. However, little is known about the effects of sacubitril/valsartan on cardiopulmonary function. Recent studies showed an improvement in exercise tolerance at 6-min walk test (6-MWT) after initiation of sacubitril/valsartan in patients with HFrEF [2,3,4]. In this clinical setting, only one study demonstrated an increase in peak oxygen consumption (VO_2_) after initiation of sacubitril/valsartan, but it was limited by a small sample size (16 patients) and a very short-term follow-up (1 month) [5].

Cardiopulmonary exercise test (CPET) is a valuable tool in HFrEF, allowing accurate assessment of patients’ functional capacity and providing prognostically relevant parameters (e.g., peak VO_2_ and minute ventilation/carbon dioxide production relationship [VE/VCO_2_ slope]) [6,7,8,9,10].

In this study, we sought to evaluate the effects of sacubitril/valsartan on prognostically significant CPET parameters in a larger population of HFrEF patients and with a longer follow-up.

## 2. Materials and Methods

### 2.1. Patient Selection and Study Design

This prospective, observational study was approved by the Institutional Research Review Boards of the Cardiovascular Rehabilitation Unit of Buccheri La Ferla Fatebenefratelli Hospital and of the Department for the Treatment and Study of Cardiothoracic Diseases and Cardiothoracic Transplantation IRCCS-ISMETT, Palermo, Italy. All patients provided informed consent. This study complies with the principles of the Declaration of Helsinki and national regulations. Sacubitril/valsartan was administered to patients with HFrEF, on top of guidelines recommended therapy [11]. Patients were included in the study in accordance with the Italian reimbursement criteria for sacubitril/valsartan: 1. symptomatic HF defined as New York Heart Association (NYHA) class II–IV, 2. left ventricular ejection fraction below 35%, as measured using echocardiography, 3. previous treatment with an individual optimal dose of angiotensin-converting enzyme inhibitor or angiotensin receptor blocker for at least 6 months, 4. systolic arterial blood pressure ≥100 mmHg, 5. serum K^+^ level <5.4 mEq/L, 6. estimated glomerular filtration rate >30 mL/min/1.73 m^2^, 7. absence of severe liver insufficiency (Child-Pugh C), and 8. no history of angioedema.

In accordance with European Society of Cardiology Prevention guidelines, patients were encouraged to have a minimum of 2.5 h a week of moderate intensity aerobic activity, in multiple bouts each lasting ≥10 min, 5 days a week [12]. Patients were not engaged in exercise-based cardiac rehabilitation, and physical activity was prescribed according to patient’s age, past habits, comorbidities, preferences, and goals.

Exclusion criteria were: 1. hospitalization for HF within 90 days before ambulatory evaluation, 2. myocardial revascularization within 180 days before ambulatory evaluation, 3. concomitant initiation of cardiac resynchronization therapy and/or percutaneous mitral valve treatment during study follow-up or in the previous 6 months, 4. congenital heart disease, and 5. inability to perform CPET.

Sacubitril/valsartan was administered according to established guidelines [11]. Up-titration was performed every 4 weeks, if tolerated by the patient. Changes in the dosage of diuretics were allowed during the study follow-up if deemed necessary. N-terminal pro-brain natriuretic peptides (NT-proBNP) serum levels were detected at baseline and at 3, 6, and 12 months.

### 2.2. CPET Protocol

Baseline CPET was performed before starting administration of sacubitril/valsartan. Serial CPETs were performed at 3, 6, and 12 months. All CPETs were performed on a cycle ergometer at 60 rpm. A ramp protocol was systematically performed: work load started at 10 watts for 2 min (warm-up) and increased by 10 watts every 60 s. Breath by breath analysis of expiratory O_2_, CO_2_, and expired volumes was performed using the Vmax^®^ 2900 metabolic cart (SensorMedics, Yorba Linda, CA, USA). Heart rate, 12 lead ECG, and oximetry (with pulse oximeter) were monitored continuously. Patients were encouraged to exercise until they felt unable to continue because of dyspnea or fatigue. The respiratory exchange ratio (RER) is the ratio between the amount of CO_2_ produced in metabolism and O_2_ used, representing a measure of exercise effort with RER > 1.05–1.10 indicating maximal effort [10]. Anaerobic threshold (i.e., the point during exercise when a switch from aerobic to anaerobic metabolism occurs) was measured using the V-slope analysis from the plot of carbon dioxide production (VCO_2_) versus VO_2_ and confirmed using ventilatory equivalents and end-tidal pressures of CO_2_ and O_2_. The rate at which VO_2_ increased per watt of work (∆VO_2_/∆Work) was calculated for the progressively increasing exercise period, beginning 1 min after work rate started to increase. ∆VO_2_/∆Work slope and VO_2_ at anaerobic threshold (AT-VO_2_) were used as a measure of muscle efficiency. The relationship between minute ventilation and carbon dioxide production (VE/VCO_2_ slope) was used as a measure of ventilatory efficiency and was calculated from 1 min after the beginning of loaded exercise up to the end of the isocapnic buffering period. Reported values of VO_2_, ventilation, and tidal volume at peak exercise are the averages over the 30 s in which the examined event occurred. Percent predicted VO_2_ represents the achieved peak VO_2_ adjusted for age, weight, and height and expressed as a percentage. We measured percent predicted VO_2_ using the equations by Wasserman and Hansen [13].

### 2.3. Statistical Analyses

Statistical analysis was performed using SAS JMP 9 software package. Continuous variables are described as mean±standard deviation, or as median and interquartile (IQ) range, in case of non-normal distribution. Categorical variables are expressed as number (percentages). Baseline and follow-up CPET parameters were compared using a Mann-Whitney U test for continuous variables and Fisher exact test for categorical variables, respectively. Changes from baseline were tested using a paired *t*-test or McNemar test, as appropriate. A *p*-value <0.05 was considered statistically significant. Nominal logistic regression was conducted to assess correlations between exercise tolerance, VO_2_, and VE/VCO_2_. A 6% increase of VO_2_ from baseline was used as a cut-off to individuate a significant improvement in VO_2_, according to current literature on this topic [10].

## 3. Results

### 3.1. Patients Characteristics

**Figure 1:**
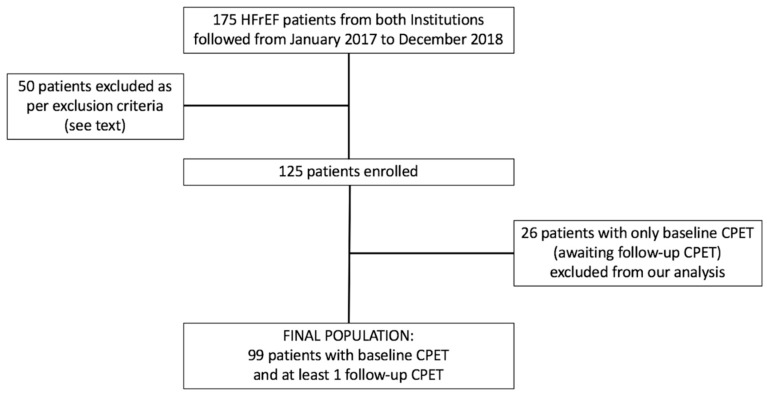
Flow chart of the study design. ARNI: angiotensin receptor-neprilysin inhibitor; CPET: cardiopulmonary exercise test; HFrEF: heart failure with reduced ejection fraction.

At present, a total of 125 patients have been enrolled and have undergone at least basal CPET. The final population for this study consisted of 99 patients for whom at least 1 follow up CPET was available (Figure 1). Baseline characteristics are listed in Table 1. Mean age was 58.7 ± 9.3 years, 86% were males, 51% had ischemic heart disease, 63% were on NYHA class II, 37% were on NYHA class III, and 17% were on atrial fibrillation. Mean left ventricular ejection fraction (LVEF) was 27 ± 6%. The starting dose of sacubitril/valsartan was 24/26 mg in 69% of patients.

At a median follow-up of 6.2 months (range 3–14.9 months), 28%, 38%, and 34% of the patients were on 24/26 mg, 49/51 mg, and 97/103 mg of sacubitril/valsartan, respectively.

Patients characteristics in the sacubitril/valsartan low and high doses cohorts are reported in Table 2.

### 3.2. CPET, NT-ProBNP, and Left Ventricular Function

Baseline and follow-up CPET results are shown in Table 3. At baseline, most patients were classified as Weber Class C and Ventilatory Class II [14]; at follow-up, we observed a 17% increase in peak VO_2_ (∆= +2.6 mL/kg/min, *p* < 0.0001), a 10.9% increase in percent predicted VO_2_ (*p* < 0.0001), and a 16% increase in O_2_ pulse (∆= +1.9 mL/beat; *p* < 0.001), and an improvement in ventilatory response with a 7% reduction in VE/VCO_2_ slope (∆= −2.4; *p* = 0.006). AT-VO_2_ increased from 11.3 ± 2.6 to 12.6 ± 3.5 mL/kg/min (*p* = 0.007); moreover, a 9% increase in ∆VO_2_/∆Work slope (∆= +0.9 mL/beat; *p* = 0.0002) and a 25% increase in exercise tolerance (∆= +18 watt; *p* < 0.0001) were obtained. At follow-up, systolic blood pressure significantly decreased from 117 ± 14 to 101 ± 12 mmHg (*p* < 0.0001) and 51 patients had a flat systolic blood pressure response during exercise (51% at follow-up versus 34% at baseline, *p* = 0.021). Of note, this did not lead to sacubitril/valsartan discontinuation in any patient.

At nominal logistic regression, increase in exercise tolerance (namely, 1-watt increase) was found to be an independent predictor of 6% improvement of VO_2_ (OR = 1.06; 95% CI: 1.03–1.10; *p* < 0.0001) at follow-up; a trend towards statistical significance was found with regard to VE/VCO_2_ slope decrease (OR = 1.02; 95% CI: 0.99–1.04; *p* = 0.057).

At follow-up, median NT-ProBNP levels decreased from 1344 (IQ range: 439–2191) to 631 pg/mL (298–1554) (*p* = 0.002).

At follow-up, mean LVEF increased from 27 ± 6 to 29.7 ± 7% (*p* < 0.0001) and left ventricular end-systolic volume decreased from 153 ± 56 to 145 ± 52 mL (*p* = 0.030).

### 3.3. CPET Results Stratified by Sacubitril/Valsartan Dosages

**Figure 2:**
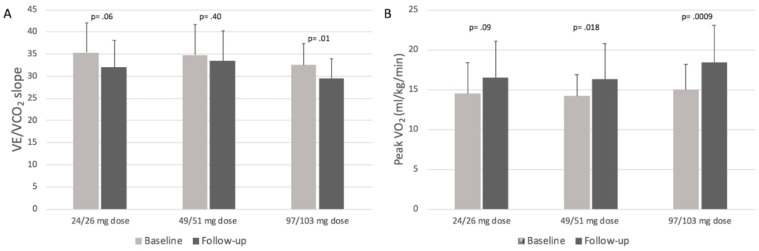
Panel **A**, VE/VCO_2_ slope variations at follow-up in patients stratified by baseline sacubitril/valsartan dosages; Panel **B**, peak VO_2_ variations at follow-up in patients stratified by sacubitril/valsartan dosages.

Peak VO_2_ variation (baseline vs follow-up) was highest in patients taking 97/103 mg of sacubitril/valsartan (∆= +3.4, *p* = 0.0009), as compared to patients taking low doses (24/26 mg) (∆= +2.0, *p* = 0.09) and medium doses (49/51 mg) (∆= +2.1, *p* = 0.018) (full data reported in Table 4). A statistically significant reduction in terms of VE/VCO_2_ slope was observed at follow-up in the subgroup of patients on the highest dose of sacubitril/valsartan (*p* = 0.01; Table 4, Figure 2A). Of note, no statistically significant differences were observed among these subgroups in terms of mean follow-up duration and baseline Peak VO_2_ (Figure 2B).

### 3.4. CPET Results Stratified by Baseline VE/VCO_2_ Slope Values

**Figure 3:**
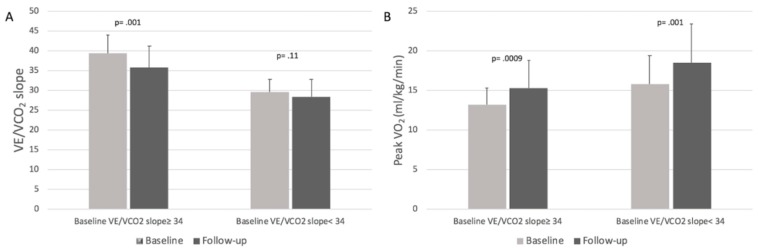
Panel **A**, VE/VCO_2_ slope variations at follow-up in patients stratified by baseline VE/VCO_2_ slope values. Panel **B**, Peak VO_2_ variations at follow-up in patients stratified by baseline VE/VCO_2_ slope values.

Patients with baseline VE/VCO_2_ ≥ 34 had a statistically significant decrease in VE/VCO_2_ slope at follow-up (39.4 ± 4.7 vs. 35.8 ± 5.4, respectively; *p* = 0.001) together with a significant increase in peak VO_2_ (13.2 ± 2.1 vs. 15.3 ± 3.5 mL/kg/min, respectively; *p* = 0.0009); patients with baseline VE/VCO_2_< 34 had a statistically significant increase in peak VO_2_ at follow-up (15.8 ± 3.6 vs. 18.5 ± 4.9 mL/kg/min, respectively; *p* = 0.001) but no significant changes in VE/VCO_2_ slope (29.6 ± 3.2 vs. 28.4 ± 4.4, respectively; *p* = 0.11) (Figure 3A,B).

Patients who reached 12-month follow-up showed the greatest reduction in VE/VCO_2_ Slope (∆= −4.7, *p* = 0.0006 for baseline versus follow-up), as compared to patients who only had 3- and 6-month follow-up (details of CPET parameters in patients stratified by follow-up duration shown in Table 5).

## 4. Discussion

CPET is a valuable tool to guide clinical decision-making and to derive prognostic information in HF patients [10,14,15,16].

In the PARADIGM-HF trial [1], sacubitril/valsartan reduced the risk of death and hospitalization for HF in patients with HFrEF, as compared to enalapril; however, little is known on how sacubitril/valsartan influences cardiopulmonary function.

To the best of our knowledge, this is the largest observational study prospectively assessing the early effects of sacubitril/valsartan on cardiopulmonary parameters in patients with HFrEF. After initiation of sacubitril/valsartan, we observed a significant improvement in the main prognostically relevant CPET parameters. To date, only one study by Palau et al. [5] showed an improvement in peak VO_2_ and VE/VCO_2_ slope in 33 HFrEF patients at 30 days follow-up after sacubitril/valsartan initiation, mostly at low doses. In our study (entailing a larger population, with a longer follow-up and including all available dosages of sacubitril/valsartan) we confirmed the significant improvement in peak VO_2_ at follow-up (∆= +2.6 mL/kg/min; *p* < 0.0001); of note, VE/VCO_2_ slope improvement started at 6 months from sacubitril/valsartan initiation and reached a statistical significant difference only at 12 months (Table 5).

The observed improvement in peak VO_2_ (+17% versus baseline) and VE/VCO_2_ slope (−7% versus baseline) at follow-up, might play a clinically and prognostically relevant role in this patient population. Swank et al. [17] reported that for every 6% increase in peak VO_2_ there is an 8% reduction in cardiovascular mortality or HF hospitalization (hazard ratio [HR] = 0.92; CI = 0.88–0.96; *p* < 0.001) and a 7% reduction in all-cause mortality (HR = 0.93; CI = 0.90–0.97; *p* < 0.001). Arena et al. [18] reported worse 1-year event-free survival from cardiac mortality (83.1% vs. 99.2%; *p* < 0.0001) and worse 1-year event-free survival from cardiac hospitalization (50.6% vs. 84.6%; *p* < 0.0001) in patients with VE/VCO_2_ slope ≥34 versus patients with VE/VCO_2_ slope <34. Furthermore, a large body of evidence confirms the prognostic relevance of VE/VCO_2_ slope values [19,20,21,22,23,24].

Notably, at follow-up, sicker patients (i.e., patients with baseline VE/VCO_2_ ≥ 34) improved both oxygen consumption and ventilatory efficiency while healthier patients (i.e., patients with baseline VE/VCO_2_ < 34) only improved oxygen consumption (Figure 3). Moreover, patients on the highest doses of sacubitril/valsartan were found to be the ones with the best functional improvement (Figure 2). These results are consistent with those reported in the PARADIGM-HF study [1].

A PARADIGM-HF post-hoc analysis by Vardeny et al. [25] demonstrates that lower doses of sacubitril/valsartan confer a similar treatment benefit over enalapril; however, patients taking low doses were associated with a higher risk of the primary events. In our study, patients taking low doses had less improvement of peak VO_2_ as compared to patients taking the highest dose; this may reflect patient frailty; indeed, patients taking low doses of sacubitril/valsartan showed lower systolic blood pressure (both at baseline and at follow-up), higher levels of NT-proBNP, increased prevalence of NYHA class III, higher furosemide dose use, lower estimated glomerular filtration rate, and a higher VE/VCO_2_ slope at baseline (details provided in Table 2 and Table 4).

Of note, exercise tolerance (namely, 1-watt increase) was found to be an independent predictor of 6% improvement of VO_2_ (OR = 1.06; 95% CI: 1.03–1.10; *p* < 0.0001) at follow-up, and a trend towards statistical significance was found with regard to VE/VCO_2_ slope decrease (OR = 1.02; 95% CI: 0.99–1.04; *p* = 0.057). It is likely that the weaker correlation with VE/VCO_2_ slope decrease might be due to the small sample size of the study population.

Sacubitril/valsartan combines the effects of angiotensin receptor blocker with neprilysin inhibition which amplify the system of natriuretic peptides and other vasoactive peptides [26,27]. However, little is known about the overall effect of vasoactive peptides on heart and lung function. In our study population, we observed an improvement of LVEF and a decrease of left ventricular end-systolic volume at follow-up. We speculate that sacubitril/valsartan might have a synergistically favorable effect on hemodynamics and muscle efficiency through reduced afterload and left ventricular filling pressure. This might result in a net improvement of exercise tolerance and performance. Of note, recent data (a longitudinal and a retrospective study) support an improvement in left ventricular ejection fraction and in left ventricular reverse remodeling after sacubitril/valsartan initiation [28,29].

We also observed an increase in peak ventilatory responses which may be secondary to the improvement of cardiac performance, allowing patients to increase ventilation without increasing the VE/VCO_2_ slope, although, at the moment, this remains speculative.

## 5. Study Limitations

This study has a number of limitations. First, we had no control group. However, the patients enrolled were hemodynamically stable and on optimized medical therapy; we may therefore consider patients at first evaluation as their own controls (versus follow-up). Importantly, since the PARADIGM-HF study has already demonstrated a relevant benefit of sacubitril/valsartan over enalapril in this setting and it is now recommended by international guidelines [11,30], denying sacubitril/valsartan to eligible patients in order to have a control group would have raised ethical issues. Conversely, selecting patients not eligible for sacubitril/valsartan as the control group, might have individuated frailer patients (i.e., with systolic arterial hypotension and more advanced chronic renal failure). Secondly, an important limitation of this study is the small sample size; nonetheless, to the best of our knowledge our work currently represents the largest series of HFrEF patients treated with sacubitril/valsartan for whom follow-up CPET parameters have been tested. Unfortunately, no data on diffusing capacity to carbon monoxide are available.

Further studies are necessary to confirm our preliminary results and to understand sacubitril/valsartan influence on cardiopulmonary function. A clinical trial evaluating the effect of sacubitril/valsartan on 6-month Exercise Tolerance in Patients with Heart Failure (NEPRIExTol) is currently ongoing (NCT03190304).

## 6. Conclusions

In this prospective observational study, administration of sacubitril/valsartan was associated with a significative improvement in exercise tolerance, peak oxygen consumption, and ventilatory efficiency at 6.2 months follow-up. Further studies are necessary to better clarify underlying mechanisms of this functional improvement.

## Figures and Tables

**Table 1:** Patient characteristics at baseline (*n* = 99).

Demographics	
Age, year, mean ± SD	58.7 ± 9.3
Female sex, no. (%)	14 (14)
SBP, mmHg, mean ± SD	117 ± 14
DBP, mmHg, mean ± SD	72 ± 10
Heart rate, beats/min, mean ± SD	67 ± 11
Body mass index, kg/m^2^, mean ± SD	28.1 ± 4.2
Medical History	
Hypertension, no. (%)	51 (51)
Diabetes, no. (%)	34 (34)
Atrial fibrillation, no. (%)	17 (17)
COPD, no. (%)	10 (10)
eGFR, mL/min/1.73m^2^, mean ± SD	67.8 ± 23.7
Nt-pro-BNP, median (IQ range)	1200 (446–2120)
LVEF (%), mean ± SD	27 ± 6
LVEDV, mL, mean ± SD	218 ± 57
LVESV, mL, mean ± SD	153 ± 56
Ischemic cardiomyopathy, no. (%)	51 (51)
Non-ischemic cardiomyopathy, no. (%)	48 (49)
NYHA functional class II, no. (%)	62 (63)
NYHA functional class III, no. (%)	37 (37)
NYHA functional class IV, no. (%)	0 (0)
Medical Therapy	
Furosemide, no. (%)	88 (89)
Furosemide dosage, mean ± SD	102 ± 105
Antialdosterone, no. (%)	87 (88)
ACE-inhibitors, no. (%)	62 (63)
ARBs, no. (%)	25 (25)
Beta-blockers, no. (%)	93 (94)
Ivabradine, no. (%)	20 (20)
Digoxin, no. (%)	7 (7)
Implantable cardioverter defibrillator, no. (%)	76 (77)
Cardiac resynchronization therapy, no. (%)	22 (22)

ACE: angiotensin-converting enzyme; ARB: angiotensin receptor inhibitor; COPD: chronic obstructive pulmonary disease; DBP, diastolic blood pressure; eGFR: estimated glomerular filtration rate (as assessed by MDRD formula); IQ: inter-quartile; LVEDV: left ventricular end-diastolic volume; LVEF: left ventricular ejection fraction; LVESV: left ventricular end-systolic volume; Nt-pro-BNP: N-terminal pro–B-type natriuretic peptide; NYHA: New York Heart Association; SBP: systolic blood pressure; SD: standard deviation.

**Table 2:** Patients characteristics in the sacubitril/valsartan low and high doses cohorts.

	Sacubitril/Valsartan 24/26 mg 28 pts	Sacubitril/Valsartan 97/103 mg 34 pts	*P* value
Baseline Characteristics			
Age, year, mean ± SD	57.8 ± 10.8	57.4 ± 8.6	0.87
Female sex, no. (%)	7 (25)	2 (6)	0.06
Ischemic cardiomyopathy, no. (%)	14 (50)	18 (53)	0.99
NYHA II, no. (%)	14 (50)	27 (79)	0.018
NYHA III, no. (%)	14 (50)	7 (21)	0.018
Diabetes, no. (%)	7 (25)	10 (29)	0.77
Atrial fibrillation, no. (%)	6 (21)	2 (6)	0.12
eGFR (MDRD), ml/min/1.73m^2^, mean ± SD	63.3 ± 21.6	72.6 ± 16.7	0.07
Furosemide dose, mean ± SD	108 ± 126	63 ± 95	0.03
Implantable cardioverter defibrillator, no. (%)	22 (78)	24 (70)	0.56
Cardiac resynchronization therapy, no. (%)	8 (28)	8 (23)	0.77
SBP, NT-pro-BNP, EDV, ESV, and LVEF (Baseline and Follow-up Data)			
SBP, mmHg, mean ± SD (Baseline)	114.3 ± 12.1	120.5 ± 14.7	0.07
SBP, mmHg, mean ± SD (Follow-up)	96 ± 11	105 ± 12	0.004
Nt-pro-BNP, median (IQ range) (Baseline)	1623.5 (477–2947)	815 (358–1929)	0.013
Nt-pro-BNP, median (IQ range) (Follow-up)	1065 (376–1739)	394.5 (195–952)	0.01
LVEDV, ml, mean±SD (Baseline)	208 ± 54	222 ± 55	0.31
LVEDV, ml, mean±SD (Follow-up)	209 ± 56	209 ± 59	0.98
LVESV, ml, mean±SD (Baseline)	147 ± 57	161 ± 48	0.29
LVESV, ml, mean±SD (Follow-up)	146 ± 57	143 ± 50	0.89
LVEF (%), mean ± SD (Baseline)	28.1 ± 5.7	28.3 ± 5.1	0.88
LVEF (%), mean ± SD (Follow-up)	28.6 ± 6.3	32.3 ± 6.6	0.026

eGFR: estimated glomerular filtration rate (as assessed by MDRD formula); IQ: inter-quartile; LVEDV: left ventricular end-diastolic volume; LVEF: left ventricular ejection fraction; LVESV: left ventricular end-systolic volume; Nt-pro-BNP: N-terminal pro–B-type natriuretic peptide; NYHA: New York Heart Association; SBP: systolic blood pressure; SD: standard deviation.

**Table 3:** Cardiopulmonary exercise test parameters (*n* = 99).

	Baseline	Follow-up	*p* value
Peak VO_2_, mL/kg/min, mean ± SD	14.6 ± 3.3	17.2 ± 4.7	<0.0001
Predicted peak VO_2_, %, mean ± SD	53.8 ± 14.1	64.7 ± 17.8	<0.0001
VE/VCO_2_ slope, mean ± SD	34.1 ± 6.3	31.7 ± 6.1	0.006
VE/VCO_2_ slope≥ 34, no. (%)	46 (46)	33 (33)	0.08
Peak RER, mean ± SD	1.12 ± 0.09	1.13 ± 0.09	0.45
Watt (Peak), mean ± SD	70 ± 22	88 ± 29	<0.0001
AT VO_2_, mL/kg/min, mean ± SD	11.3 ± 2.6	12.6 ± 3.5	0.007
Predicted AT VO_2,_ %, mean ± SD	42.3 ± 11.5	47.2 ± 12.5	0.009
AT undetectable, no. (%), mean ± SD	16 (16)	9 (9)	0.19
O_2_pulse (ml/beat)	11.5 ± 3.0	13.4 ± 4.3	0.0007
∆VO_2_/∆work, mL/min/watt, mean ± SD	9.2 ± 1.5	10.1 ± 1.8	0.0002
Peak ventilation, L/min, mean ± SD	48.7 ± 12.7	59.3 ± 18.9	<0.0001
Peak tidal volume, L, mean ± SD	1.57 ± 0.43	1.75 ± 0.53	0.009
Peak Respiratory rate, b/m, mean ± SD	30.5 ± 6.7	33.3 ± 7.2	0.006
Ventilatory Oscillation, no. (%)	31 (31)	19 (19)	0.07

AT: anaerobic threshold; RER: respiratory exchange ratio; SD: standard deviation; VE/VCO_2_: minute ventilation/carbon dioxide production ratio; VO_2_: oxygen consumption.

**Table 4:** Cardiopulmonary exercise test parameters stratified by sacubitril/valsartan dosages.

	Baseline	Follow-up	*p* value
Peak VO_2_, mL/kg/min, mean ± SD			
24/26 mg dose (28 pts)	14.5 ± 3.9	16.5 ± 4.6	0.09
49/51 mg dose (37 pts)	14.2 ± 2.7	16.3 ± 4.5	0.018
97/103 mg dose (34 pts)	15 ± 3.2	18.4 ± 4.7	0.0009
Predicted peak VO_2_, %, mean ± SD			
24/26 mg dose (28 pts)	54 ± 12.9	62.1 ± 14.1	0.029
49/51 mg dose (37 pts)	53.8 ± 13.9	61.9 ± 16.6	0.02
97/103 mg dose (34 pts)	53.6 ± 15.6	68.6 ± 20.6	0.001
VE/VCO_2_ slope, mean ± SD			
24/26 mg dose (28 pts)	35.3 ± 6.8	32 ± 6.1	0.06
49/51 mg dose (37 pts)	34.8 ± 6.9	33.4 ± 6.9	0.4
97/103 mg dose (34 pts)	32.5 ± 4.9	29.5 ± 4.5	0.01
O_2_ pulse, ml/beat, mean ± SD			
24/26 mg dose (28 pts)	11.4 ± 3.1	12.8 ± 4.3	0.016
49/51 mg dose (37 pts)	11 ± 3.1	12.3 ± 3.9	0.12
97/103 mg dose (34 pts)	12.2 ± 2.8	14.9 ± 4.4	0.003
∆VO_2_/∆work, mL/min/watt, mean ± SD			
24/26 mg dose (28 pts)	9.1 ± 1.3	9.7 ± 2.2	0.24
49/51 mg dose (37 pts)	9 ± 1.6	9.9 ± 1.8	0.028
97/103 mg dose (34 pts)	9.3 ± 1.5	10.5 ± 1.4	0.001

SD: standard deviation; VE/VCO_2_: minute ventilation/carbon dioxide production ratio; VO_2_: oxygen consumption.

**Table 5:** Cardiopulmonary exercise test parameters in patients stratified by follow-up duration.

	Baseline	Follow-up	*p* value
Peak VO_2_, mL/kg/min, mean ± SD			
3 months (24 pts)	15.3 ± 3	16.9 ± 4.1	0.12
6 months (40 pts)	14.8 ± 3.6	17.1 ± 5	0.02
12 months (35 pts)	13.8 ± 3	17.3 ± 4.6	0.0006
Predicted peak VO_2_, %, mean ± SD			
3 months (24 pts)	54.9 ± 9.5	61.3 ± 13.1	0.06
6 months (40 pts)	56.7 ± 14.5	66.9 ± 17.2	0.0005
12 months (35 pts)	49.7 ± 15.6	63.4 ± 20.4	0.002
VE/VCO_2_ slope, mean ± SD			
3 months (24 pts)	33.7 ± 6.2	33.4 ± 7.8	0.9
6 months (40 pts)	33.3 ± 6.6	31.4 ± 6	0.19
12 months (35 pts)	35.4 ± 6	30.7 ± 4.8	0.0006
O_2_ pulse, ml/beat, mean ± SD			
3 months (24 pts)	12.3 ± 3.3	13.0 ± 4.2	0.52
6 months (40 pts)	11.3 ± 3.2	13.3 ± 4.3	0.023
12 months (35 pts)	11.2 ± 2.5	13.6 ± 4.5	0.007
∆VO_2_/∆work, mL/min/watt, mean ± SD			
3 months (24 pts)	9.4 ± 1.4	10.3 ± 1.7	0.09
6 months (40 pts)	9.3 ± 1.2	10.1 ± 2.1	0.042
12 months (35 pts)	8.8 ± 1.7	9.9 ± 1.7	0.007

SD: standard deviation; VE/VCO_2_: minute ventilation/carbon dioxide production ratio; VO_2_: oxygen consumption

## Abbreviations List

AT-VO_2_	oxygen consumption at anaerobic threshold
CPET	cardiopulmonary exercise test
HF	heart failure
HFrEF	heart failure with reduced ejection fraction
LVEF	left ventricular ejection fraction
NYHA	New York Heart Association
NT-proBNP	N-terminal pro-brain natriuretic peptides
RER	respiratory exchange ratio
VE/VCO_2_	ventilation/carbon dioxide production relationship
VO_2_	oxygen consumption
